# The Mediating Role of History of Heart Disease in the Relationship Between COVID-19 and Dementia

**DOI:** 10.1093/geroni/igaf122.2311

**Published:** 2025-12-31

**Authors:** Reza Amini, Jahanvi Patel

**Affiliations:** University of Michigan-Flint, Flint, Michigan, United States; University of Michigan-Flint, Flint, Michigan, United States

## Abstract

This study used data from the National Health and Aging Trends Study (NHATS) spanning 2011-2012 to explore the long-term health impacts of COVID-19, with a focus on cardiovascular and cognitive outcomes. We hypothesized that COVID-19 exacerbates the risk of cardiovascular disease (CVD), which then leads to increased hospitalizations and cognitive decline. Employing Structural Equation Modeling (SEM), we analyzed the interrelationships among COVID-19 severity, history of CVD, hospital stays, and dementia across two waves. Our results confirm that severe COVID-19 significantly escalates the risk of a history of CVD. These conditions serve as crucial mediators that not only increase the probability of hospitalization but also contribute to the risk of dementia. The SEM model demonstrated that COVID-19 indirectly impacts cognitive function through its effect on cardiovascular health, with significant pathways linking heart disease to higher hospitalization rates and increased risk of dementia (Standardized β = 0.161, p < 0.001 for hospitalization; β = 0.037, p = 0.030 for cognitive decline). The optimal fit of the model (RMSEA ∼0.00, CFI ∼1.00) indicates an excellent replication of the data structure, affirming the model’s accuracy in capturing the relationships among the study variables. These findings highlight the imperative of cardiovascular monitoring in COVID-19 recovery plans and suggest that addressing cardiovascular and cognitive health in tandem may enhance outcomes for COVID-19 survivors. This research contributes to a deeper understanding of the pandemic’s multifaceted impact on public health and underscores the necessity for integrated healthcare strategies that consider the interplay between infectious diseases and chronic conditions.

